# Dynamical complexity and computation in recurrent neural networks beyond their fixed point

**DOI:** 10.1038/s41598-018-21624-2

**Published:** 2018-02-20

**Authors:** Bicky A. Marquez, Laurent Larger, Maxime Jacquot, Yanne K. Chembo, Daniel Brunner

**Affiliations:** 1 FEMTO-ST Institute, CNRS & Univ. Bourgogne Franche-Comté, 15B Avenue des Montboucons, Besançon Cedex, 25030 France; 2 GeorgiaTech-CNRS Joint International Laboratory [UMI 2958], Atlanta Mirror Site, School of Electrical and Computer Engineering, 777 Atlantic Dr NW, Atlanta, GA 30332 USA

## Abstract

Spontaneous activity found in neural networks usually results in a reduction of computational performance. As a consequence, artificial neural networks are often operated at the edge of chaos, where the network is stable yet highly susceptible to input information. Surprisingly, regular spontaneous dynamics in Neural Networks beyond their resting state possess a high degree of spatio-temporal synchronization, a situation that can also be found in biological neural networks. Characterizing information preservation via complexity indices, we show how spatial synchronization allows rRNNs to reduce the negative impact of regular spontaneous dynamics on their computational performance.

## Introduction

Random recurrent neural networks (rRNNs) are popular models to investigate basic principles of information processing inside the human brain. Although brain connectivity cannot be assumed to be fully random^1,2^, there is experimental support suggesting that some parts of the brain are described by stochastic architectures. For example, in insect’s olfactory systems the odour recognition process is performed by olfactory receptor neurons with structureless (random) synaptic connections^3,4^. The random weights of these networks also serve as tools for dimensionality reduction^5,6^. In models investigating such situations, the synaptic neural links, defined by the elements of a random matrix, would follow a Gaussian distribution^7–10^.

Despite the fact that interactions inside rRNNs are governed according to random coupling, these networks can still achieve highly coherent collective behavior^1,11–13^. Under such conditions they can experience phase synchronized dynamics, which have been identified to play an important role in biological memory processes^14^, neural communication^15–17^ and plasticity^18–21^. Synchronization therefore is a relevant mechanism in biological neural networks. Such synchronous phenomena take the form of regular spatio-temporal patterns, showing the presence of self-organization. These patterns can also be found in homogeneous^22^ as well as in heterogeneous artificial neural networks^23,24^. Dynamics of the here discussed rRNNs can be tuned via a single parameter, which typically results in a bifurcation phenomena as a route to chaos^25–27^. Furthermore, this transition in their dynamical properties possibly influences the network’s spatio-temporal synchronization.

Besides their function as model systems in biological neuroscience, rRNNs have been widely studied in the machine learning community due to their excellent computational properties. In rRNNs, special attention was given to computation at the transition from a steady state to chaotic dynamics, defined as the edge of chaos. Essential for solving complex tasks, operating a network at the edge of chaos ensures a high susceptibility to perturbations and hence its excitability^25–29^. Beyond the edge of chaos, the network typically experiences non-regular dynamics even in the absence of an external stimulus. Such spontaneous dynamics are considered a nuisance as they disrupt the causality between input and network state. Nevertheless, information processing might not depend on individual node dynamics. In other words: local details of an autonomous rRNN’s dynamical state might be of secondary importance, as long as the network as a whole can preserve the information content of the injected signal. Global dynamical properties and their influence on computation therefore deserve a closer inspection.

In this work, we study a rRNN predicting a chaotic time series. Identifying each node as a spatial-position, the network’s state variable can be interpreted in terms of spatio-temporal dynamics. Motivated by the impact of spatio-temporal dynamical properties, we particularly focus on beyond fixed point operation. We employ a variation to classical rRNNs^25–27,30^ by using nodes with a sinusoidal activation function. A broad range of autonomous dynamics are the consequence, among which we most importantly find multiple non-fixed point states with surprisingly high computational performance. We show that spatial synchronization between nodes and bifurcation point play essential roles in information processing. The underlying mechanisms are analysed based on the mutual information between each node of rRNN and the input time signal, as well as the rRNN’s maximal Lyapunov exponent. Our choice of system is directly motivated by its randomness: we can exclude structural modifications induced by learning being the cause behind spatial synchronization.

## Results

### Random recurrent neural networks

**Figure 1:**
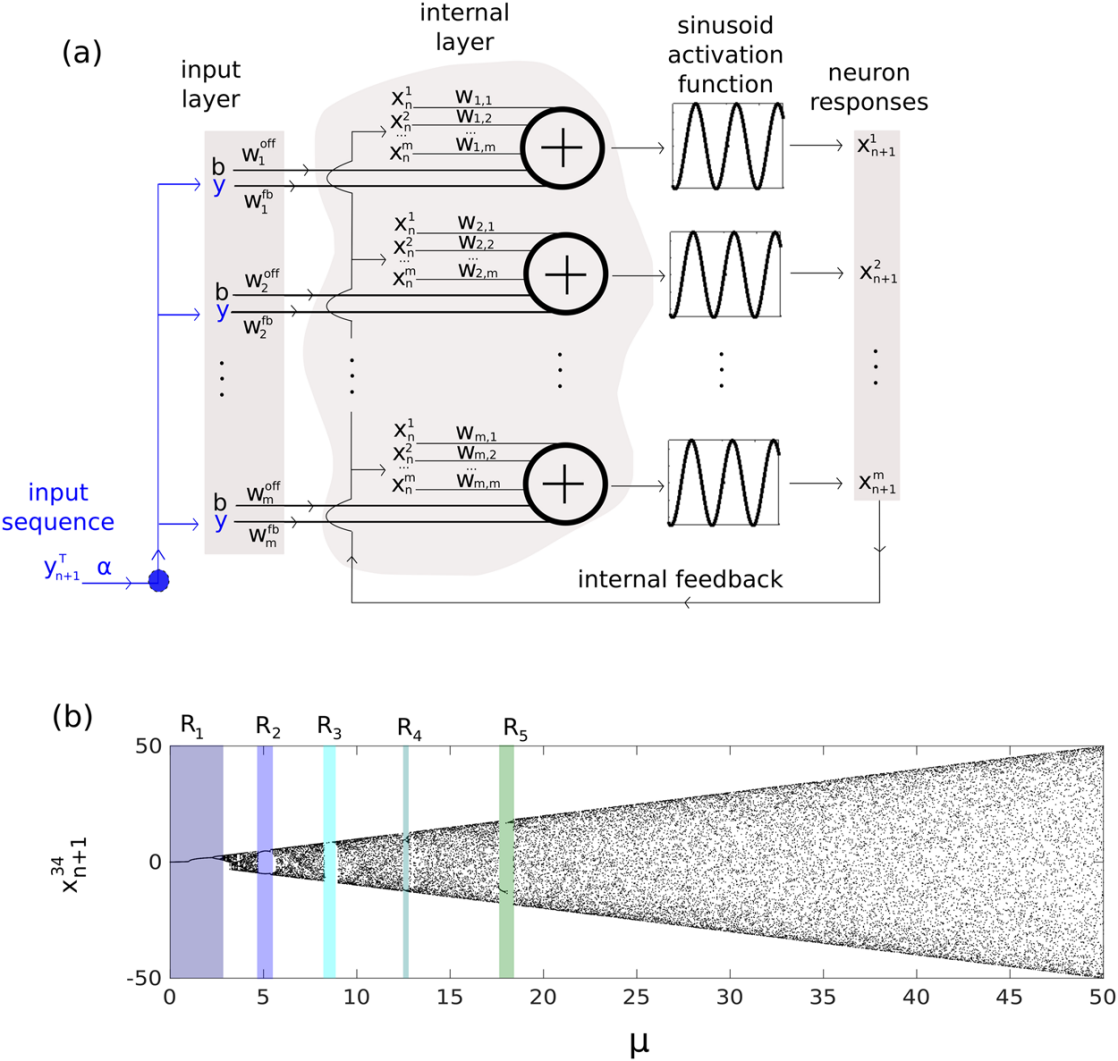
Neural Network and a typical node Bifurcation. (**a**) Schematic illustration of the rRNN structure with sinusoid activation function. (**b**) Bifurcation diagram of node 34 (${x}_{n+1}^{34}$), with regions of non-chaotic behavior: R_1_, R_2_, R_3_, R_4_, and R_5_.

Our rRNN consists of a set of *N* = 500 nodes in state *x*_*n*_, internally connected via a random, uniformly distributed internal weight matrix *W* of dimensionality *N* × *N*. The resulting random networks have a temporal evolution governed by

1 $${{\bf{x}}}_{n+1}=\mu \,\sin (W\cdot {x}_{n}+{W}^{off}\cdot b+{W}^{fb}\cdot \alpha {y}_{n+1}^{T}),$$

where {*W*, *W*^*off*^, *W*^*fb*^} are matrices defining the random weight connectivity of the rRNN for the network itself, *b* = 0.2 the offset operating points at each node, and the input layer connectivity with the signal $\alpha \cdot {y}_{n+1}^{T}$ (*α* the input scaling), respectively. *μ* is the feedback amplification. The connectivity matrix *W* is constructed with 500 × 500 random, uniformly distributed coefficients in [0, 1], from a matrix with connectivity 0.99. The rRNN is schematically illustrated in Fig. 1(a), where nodes (symbol ⊕) add and nonlinearly transform all inputs $\{{{\bf{x}}}_{n},b,{y}_{n+1}^{T}\}$ according to random weights {*W*, *W*^*off*^, *W*^*fb*^}.

In its bifurcation diagram this neural network experience multiple windows of regular dynamics. In Fig. 1(b), we show a single node’s bifurcation diagram. The windows of regular dynamics appear at distinct values which are highly comparable for all nodes. Dynamics are obtained for *μ* in [0, 50] and for a randomly chosen node, i.e. node 34 (${x}_{n+1}^{34}$) of the autonomous system (*α* = 0). Non-chaotic regimes can be found in several regions: R_1_, R_2_, R_3_, R_4_, and R_5_, for *μ* ∈ [0.1, 2.8], *μ* ∈ [4.8, 5.4], *μ* ∈ [8.5, 8.9], *μ* ∈ [12.4, 12.7], and *μ* ∈ [17.6, 18.4], respectively. These regions are part of the multistability present in this particular rRNN, consequence of the sinusoidal nonlinear function. Standard rRNNs typically have a hyperbolic tangent as their nonlinear function^30^. In that case, a steady state only exists for *μ* < 1.4. However, once it bifurcates there are no additional steady state windows that can be used for investigating their information processing properties, i.e. the network’s dynamic falls in a chaotic regime. In such a system one could not compare results obtained under similar dynamical states. As we intent to investigate general properties for computation in non-steady state systems, we opted for our modified rRNN.

**Figure 2:**
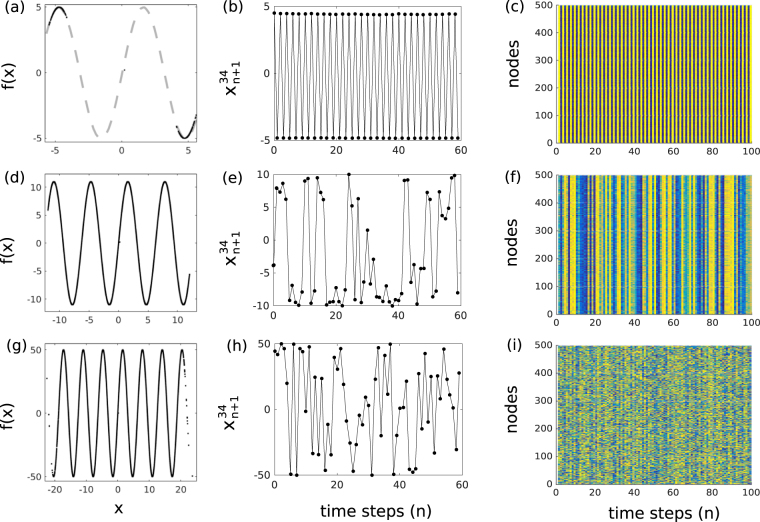
Dynamical evolution of the rRNN. Nonlinear response of nodes with *f*(*x*) = sin(*x*) (left column), time series recorded for node ${x}_{n+1}^{34}$ (center column), and spatio-temporal evolution of the rRNN (right column): (**a**–**c**) *μ* = 5, (**d**–**f**) *μ* = 10, and (**g**–**i**) *μ* = 50. All dynamics obtained for the autonomous system (*α* = 0).

Autonomous rRNN dynamics (*α* = 0) for different values of the bifurcation parameter *μ* are shown in Fig. 2. The left column shows the functional input-output relationship of Eq. (1) for node 34. The central column displays exemplary individual time series for the same node, referred to as local dynamics, while the right column shows the dynamical state of the full rRNN. For *μ* = 5 (regime R_2_) the node state is symmetrically concentrated along the nonlinear function’s extrema, see panel (a) of Fig. 2. The resulting dynamics of ${x}_{n+1}^{34}$ and the full networks state ***x***_*n*+1_ is shown in Fig. 2(b,c), respectively. Autonomous dynamics of ${x}_{n+1}^{34}$ are therefore periodic, and according to Fig. 2(c) such local periodic dynamics strongly synchronize across the rRNN. When increasing bifurcation parameter *μ* to 10, dynamics span an increasing number of the nonlinear function’s periods, see Fig. 2(d). The consequence is a local dynamical state with considerably higher complexity, see Fig. 2(e). Yet, synchronization between individual nodes still proofs to be robust. According to Fig. 2(f), regular spatial patterns are still present and synchronization across the network is preserved despite the chaotic dynamics of individual nodes^31^. Finally, when further increasing *μ* to 50, dynamics spanning up to 14 extrema (Fig. 2(g)) result in hyper-chaotic node responses, see Fig. 2(h). As illustrated in Fig. 2(i), only for such large bifurcation values the regular spatio-temporal structures across the network have vanished and synchronization is lost.

### Mitigating autonomous dynamics by learning

All previously discussed dynamical properties have been exclusively obtained in the absence of an external stimulus. However, as an information processing system, the rRNN realizes computation on the bases of rich dynamical responses to external, i.e. sensory input. We therefore activate the rRNN’s input by setting *α* = 0.8 and investigate its dynamics when fed by a chaotic time series. We add an output layer that provides the computational result according to

2 $${y}_{n+1}^{out}=\,\tanh ({W}_{op}^{out}\cdot {{\bf{x}}}_{n+1}\mathrm{).}$$

A divergence of the system’s output *y*^*out*^ for large *μ* is avoided by limiting the range of *y*^*out*^ through the hyperbolic tangent in Eq. (2). The output weight vector *W*^*out*^ is calculated according to a supervised learning rule based on a teacher/target signal $\alpha {y}_{n+1}^{T}$, Eq. (3).

**Figure 3:**
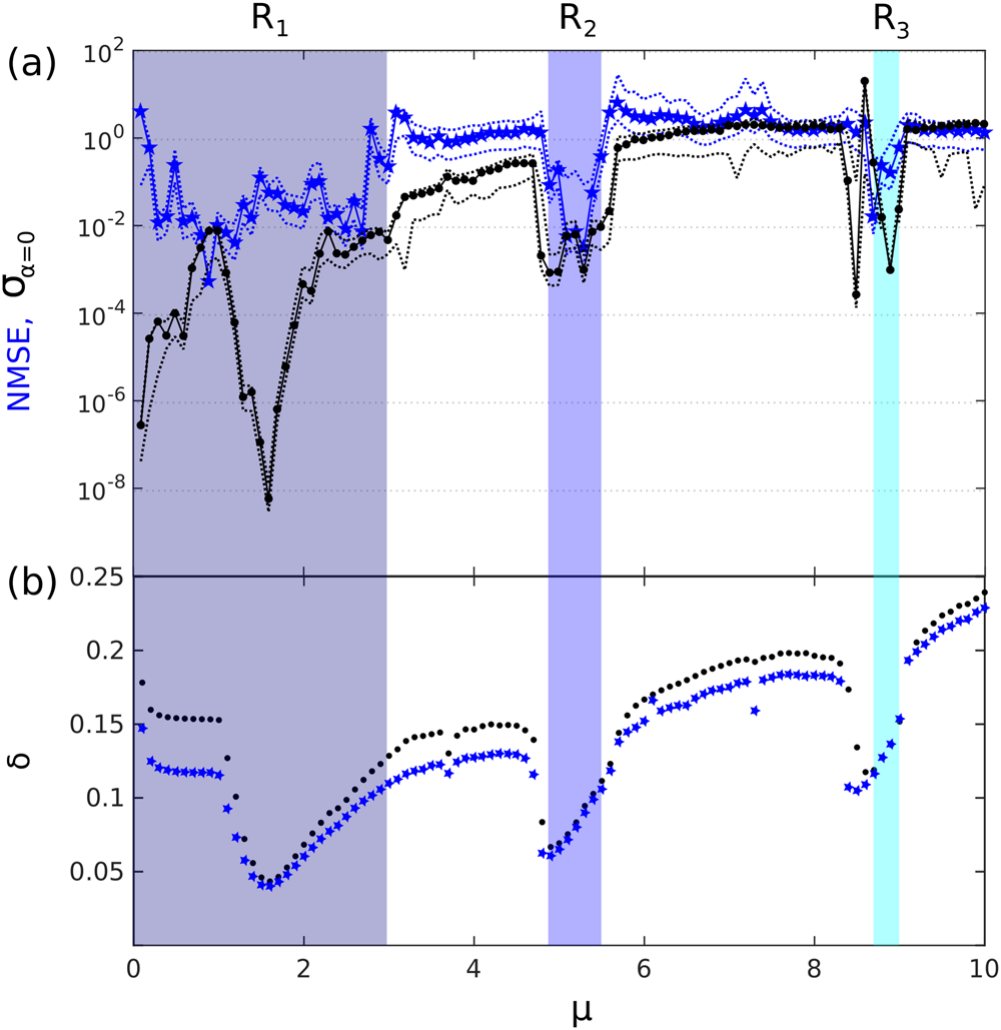
rRNN prediction performance, noise supression and synchronization. (**a**) Average NMSE as a function of *μ* (blue stars), and the noise-like standard deviation in rRNN output *σ*_*α*=0_ for the autonomous system (black circles) with error-limits presented by envelops of dotted-curves. (**b**) Average synchronization error across the rRNN for the driven (*α* = 0.8) and autonomous system (*α* = 0) in black dots and blue stars, respectively. Data was averaged over 100 different realizations of the rRNN.

Once trained, the input signal is replaced by the network’s own output^30^ ${y}_{n+1}^{T}={y}_{n+1}^{out}$ in Eq. (1), and the system autonomously approximates dynamics learned from the teacher system, here the Mackey-Glass (MG) sequence from Eq. (7)^32^. Computational performance is determined after a free evolution of 35 time steps, twice the time-delay of the MG sequence (*τ*_*m*_ = 17 in Eq. (7))^32,33^. In Fig. 3(a) the prediction NMSE (Eq. (4)) is shown for 0 < *μ* ≤ 10. At each *μ* we repeated the previously introduced training procedure. The optimal performance (NMSE = 5.5 × 10^−4^) is found for a very narrow regime around *μ* = 0.9, which comes at no surprise as it corresponds to the often employed computation close to the self organizing criticality^25^. However, additionally we identify multiple broader regions of acceptable performance with a prediction error of roughly NMSE ≈10^−2^. A comparison to the rRNN’s bifurcation diagram of Fig. 1(b) reveals that these regions directly correspond to R_1_, R_2_ and R_3_, where rRNN dynamics are regular and nodes are synchronized. Regimes *R*_4_ and *R*_5_ are not treated in our analysis since small perturbations result in their destabilization, driving the rRNN instantaneously into the next chaotic regime. In all other regions the error is orders of magnitude higher.

As previously introduced, in the self-driven mode the rRNN’s output becomes its own input. If not suppressed, perturbation-like autonomous rRNN dynamics can therefore freely propagate through the system due to this recurrent input/output relationship. This raises the question as to how the network can mitigate internal dynamics so efficiently. The answer lies within the learning process. We demonstrate this by creating the rRNN’s output ${y}_{n+1}^{out}$ for *α* = 0, however using the *W*^*out*^ previously learned for approximating ${y}_{n+1}^{T}$ for the driven system at *α* = 0.8. From the resulting signal we discard the first 10 data points to avoid possible transient behavior. From the remaining 25 samples we calculate the average output amplitude variation *σ*_α=0_ via Eq. (5). This measure *σ*_*α*=0_ evaluates the output weights’ performance for suppressing autonomous dynamics, and the black data in Fig. 3(a) demonstrates how intricate *σ*_*α*=0_ and the NMSE are related. Within regions R_1_, R_2_ and R_3_, learning efficiently separates autonomous dynamics from transients induced by the rRNN’s input. As revealed by the low values of *σ*_*α*=0_, the rRNN can there approximate ${y}_{n+1}^{T}$ well because the impact of autonomous rRNN dynamics on *y*^*out*^ is strongly reduced. As a consequence, perturbations not present in the training data can be isolated from dynamics in the target data.

In general, the spontaneous internal activity of each node has an impact on the collective evolution of the network. As illustrated in the right column of Fig. 2, this recurrent network architecture can cause different levels of dynamical diversity through out the rRNN, which in turn potentially influences the propagation of information within the network. The spatio-temporal evolution of the rRNN can be quantified by the spatial synchronization between the nodes. The standard deviation, described by Eq. (6), globally measures spatial synchronization between nodes^24^. Figure 3(b) shows how uniformly synchronous the nodes are depending on the value of *μ* when the network presents autonomous (black dots) and driven activity (blue stars). The nodes of the rRNN are less synchronized in the non-regular windows when the network is perturbed by an external signal. In regions R_1_, R_2_ and R_3_, however, synchronization is nearly preserved in both cases. As demonstrated by Fig. 3(b), the global rRNN synchronization error significantly decreases in regions R_1_, R_2_ and R_3_. Thus, the dependence of synchronization *δ* on bifurcation parameter *μ* is highly comparable for, both, the driven and the autonomous system. Region R_1_ can clearly be separated into two sections. For *μ* ≤ 1 node responses consists of constant states; the system is operating in the linear section of the nonlinear function. For *μ* > 1 the network states also cover the nonlinear function’s extrema and nodes start evolving in synchrony. Region R_3_ is significantly more sensitive to *μ* when compared to R_1_ and R_2_. This is due to the proximity to parameters resulting in chaotic dynamics. Such a sensitive operating point is less recommendable when for example we use a noisy hardware or biological rRNN for prediction. In fact, in R_3_ we find that the nodes are in steady states with small or vanishing amplitude dynamics, however not as well synchronized as in R_1_, and R _2_. The comparison between panels (a) and (b) of Fig. 3 highlights the importance of spatial synchronization for good prediction performance. Data shown in Fig. 3 shows the statistical average obtained from 100 realizations of *W*.

**Figure 4:**
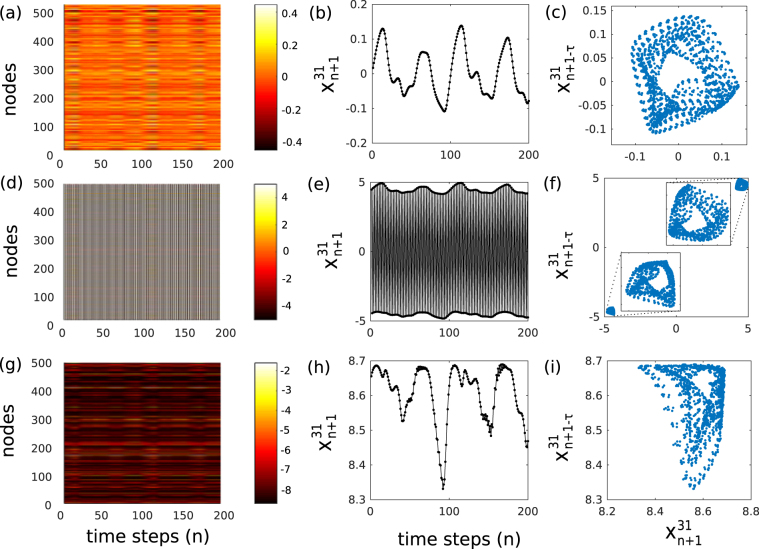
Spatio-temporal evolution of the rRNN (left column), time series recorded for node ${x}_{n+1}^{31}$ (center column), and 2D projections of the reconstructed time series from node 31 (right column). (**a**–**c**) *μ* = 0.8, (**d**–**f**) *μ* = 5, and (**g**–**i**) *μ* = 8.7. All dynamics obtained for the driven system (*α* = 0.8).

An extensive qualitative analysis of the dynamics associated with good prediction performance is shown by Fig. 4. For *μ* = 0.8, Fig. 4(a) shows the spatio-temporal plot of all nodes when the input is injected in steady state regime R_1_. Column-shaped patterns throughout the entire spatio-temporal plot are induced by the external data and therefore indicate information preservation. One example of how input information is preserved within the rRNN is shown by Fig. 4(b), where the randomly chosen node 31 (${x}_{n+1}^{31}$) shows a nonlinearly transformed version of the input. For a geometrical illustration of the information carried by the node, we illustrate the system’s dynamic by reconstructing the attractor of node 31 through Takens embedding Theorem^34^. We used embedding parameters of delay *τ* = 12 and dimensions *D* = 4 which are the ones obtained for delay embedding the original MG attractor^35^, also see Methods section. A 2D projection of resulting state space is shown by Fig. 4(c), which is qualitatively comparable with the structure of the chaotic MG attractor shown in the Methods section.

At *μ* = 5 (regime R_2_) we find the previously described periodic oscillations. The spatio-temporal plot of the driven rRNN shows once again a constant phase relation across all nodes, see Fig. 4(d). Spatio-temporal features corresponding to the input timetrace masked by the autonomous oscillations. Consequently, the closer inspection of individual node evolutions shows that the spontaneous rRNN internal dynamics are still present, see Fig. 4(e). We find that node dynamics consist of two contributions. Large amplitude oscillations at fast timescales correspond to the autonomous dynamics, while nonlinear transients induced by the input information are encoded in the slowly varying envelope. Such specific and well separated time scales are a requirement for suppressing the crosstalk of autonomous dynamics to *y*^*out*^. Combined with Fig. 4(f), the impact of regular autonomous rRNN dynamics becomes clear. The fast autonomous oscillations separate the node’s attractor into two regions of its state space. Within each of these regions, the local attractor again resembles the one of the injected MG sequence. As such, removing this division by training corresponds to *σ*_*α*=0_, and the system should still be able to approximate the target attractor^36^. Periodic dynamics ensure the discussed separation between timescales, while synchronization minimizes the resources learning has to dedicate for their suppression, leaving more freedom for optimizing prediction performance.

Upon increasing the bifurcation parameter to *μ* = 8.7 (regime R_3_), the collective dynamics shown by Fig. 4(g) have similarities to the one shown in panel (a), even experiencing a degree of synchronization. In this case, the node’s responses to the external information is perturbed by irregularly appearing, noise-like epochs, see Fig. 4(h). The fixed point of R_3_ is a quasi-steady state, yet due to the narrow width of R_3_ the rRNN is forced outside this stability window even by small fluctuations which in turn can induce noise like epochs. Figure 4(i) shows the effect of the noise on the reconstructed attractor. The noise strongly distorts the node responses away from the MG attractor. As induced by noise-like epochs, these distortions strongly hamper the determinism in the rRNN’s response to the injected information.

### Preservation of information in destabilized rRNNs

Spontaneous dynamics in the rRNN therefore result in distortions of its response. At this point it is important to recall that the input sequence is chaotic, yet by no means random. It is the result of complex, yet causal deterministic processes. Predicting such a signal therefore demands these causal relationships to be preserved within the neural network’s dynamical state, providing a functional relationship to currently and previously injected information. For low error prediction it is therefore an essential condition that the network can serve as carrier and short term storage of injected information. Synchronization is not sufficient to estimate if a rRNN complies with this condition. Quantifying the information content preserved within the rRNN when stimulated by an input, we calculate the mutual information (MI) between the rRNN and the input signal. This provides an estimation of how well the network is able to maintain the input information content^25^, and hence is capable to capitalize from these internal causal relationships for computation.

**Figure 5:**
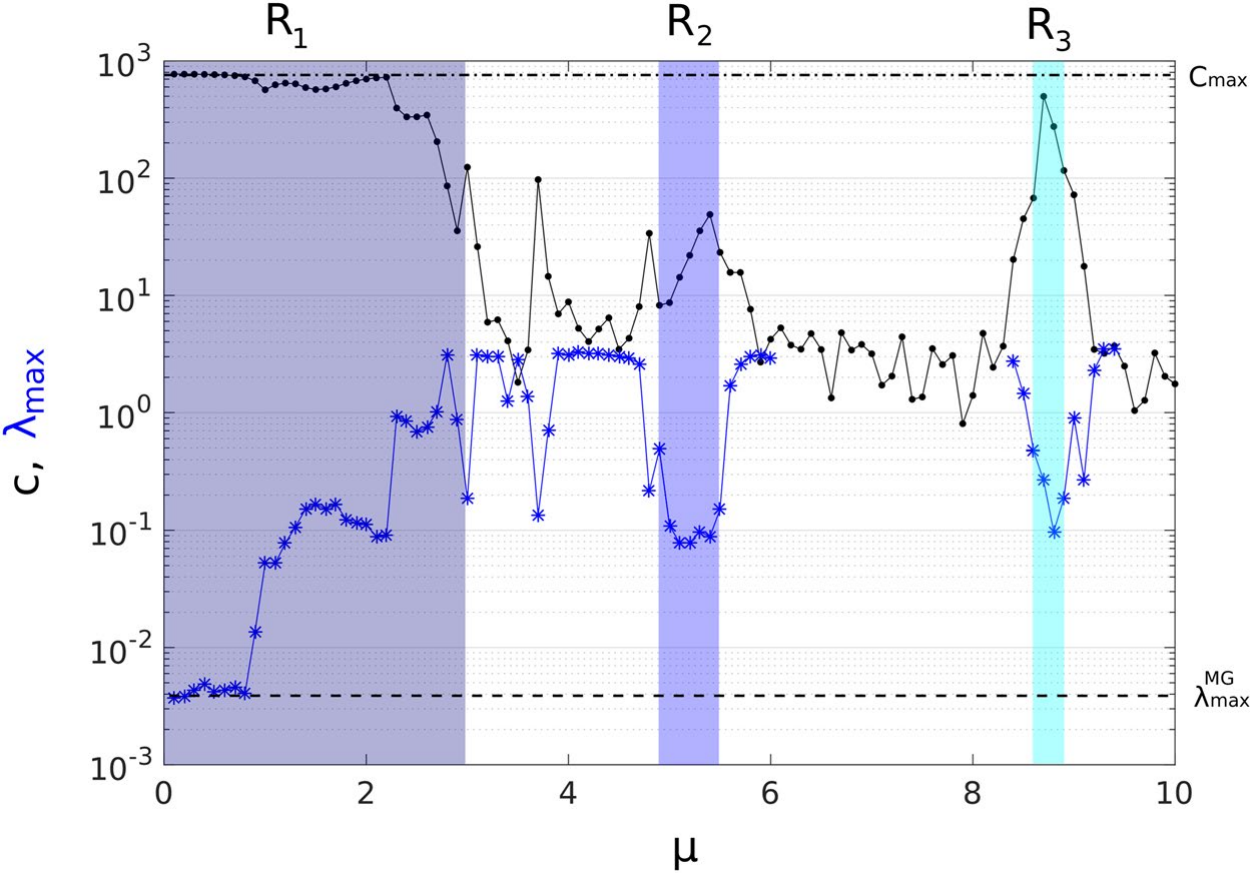
rRNN mermory capacity and maximal Lyapunov exponent. Memory capacity C (black dots), and maximal Lyapunov exponent *λ*_*max*_ (blue stars) of the rRNN, as functions of *μ*. Sections without *λ*_*max*_ values exhibit hyper-chaotic dynamics with more than one positive Lyapunov exponent. The dashed/dotted line indicates ${\lambda }_{max}^{MG}/{C}_{max}$, respectively.

We consequently evaluate the network by estimating the memory capacity *C* via the mutual information between each node and the input, see Eq. (8), and maximal Lyapunov exponent *λ*_*max*_, as functions of the bifurcation parameter. In Fig. 5 we show the rRNN’s *C* and *λ*_*max*_ as black dots and blue stars, respectively. As during our previous analysis, we find that regimes R_1_, R_2_, and R_3_ show their capabilities to accurately preserve previous input information. In steady state regimes R_1_ and R_3_ the memory capacity *C* is higher than in R_2_, where the rRNN’s spontaneous behavior is periodic in general. Our Lyapunov component analysis reveals that *λ*_*max*_ is kept small inside R_1_, R_2_, and R_3_ due to their non-chaotic spontaneous features. In fact, for *μ* ≤ 1 in R_1_, *λ*_*max*_ of the network approaches with the one estimated for the input signal (${\lambda }_{max}^{MG}\sim 3.6\times {10}^{-3}$), indicated by the dashed line in Fig. 5. For *μ* > 1, oscillatory, spontaneous rRNN’s dynamics are combined with the injected input information. As the internal dynamics of the rRNN begin to exert influence over dynamics induced by the MG input, *λ*_*max*_ starts increasing accordingly. This behavior agrees well with the decrease of memory capacity, where the internal rRNN’s dynamics will modify the probability distribution of the nodes. This demonstrates a strong correlation between the decline of spatio-temporal synchronization and the reduction in the system’s memory capacity to approximate the deterministic, functional relationship of the prediction task.

## Conclusion

Unlike neural networks run on a data-center, the human brain is not a special purpose computing machine, but parameters will most likely be optimized according to a compromise between partially competing demands. We therefore demonstrated that information preservation and synchronization inside a random network allow good prediction performance at parameters where learning in biological neural networks benefits. Based on a rRNN with a periodic nonlinear function we compare various regions of regular dynamics and highlight their importance of spatial synchronization upon prediction performance, mutual information and the stability of the neural network. Synchronization between nodes plays an essential role, but it is not sufficient to understand how information processing is successful in a rRNN beyond its fixed point. On the contrary, when linear regression is used to realize supervised learning, a causal relation between processed information and target is required.

We describe a rRNN predicting the future time-steps of a chaotic trajectory. Our results illustrate the importance of information flow, divergence and the suppression of signal components not present in the training data set. The rRNN’s damped autonomous deviation *σ*_*α*=0_, mutual information *MI* and maximal Lyapunov exponent can be seen as complexity indicators for interpreting neural networks based on dynamical systems. Other than for the oscillatory state, chaotic responses were not capable to maintain important features of the input dynamic, resulting in a low prediction performance. Finally, delay systems exploiting an identical nonlinearity have been reported^37,38^ and it would be of interest to investigate such hardware systems based on the here introduced methodology.

## Methods

### Training of the rRNN

For the training step we use 2000 values from the MG system and *α* = 0.8^30,32,33^. The training target is equivalent to the input signal, shifted by a single time step. Via the teacher we estimate the optimal output weight vector ${W}_{op}^{out}$

3 $${W}_{op}^{out}=\mathop{{\rm{\min }}}\limits_{{W}^{out}}\Vert \tanh ({W}^{out}\cdot {{\bf{x}}}_{n+1})-{y}_{n+1}^{T}\Vert ,$$

via its pseudo-inverse according to singular value decomposition. Equation (3) therefore minimizes the error between output tanh(*W*^*out*^ ⋅ x_*n* +1_) and teacher ${y}_{n+1}^{T}$. As training error measure we use the *normalized mean squared error* (NMSE) between output ${y}_{n+1}^{out}$ and target signal ${y}_{n+1}^{T}$, normalized by the variance of teacher signal ${y}_{n+1}^{T}$:

4 $$NMSE=\frac{1}{M}\frac{\sum _{n=1}^{M}{({y}_{n+1}^{out}-{y}_{n+1}^{T})}^{2}}{{\sigma }^{2}({y}_{n+1}^{T})},$$

where *σ* is the standard deviation.

### Statistical amplitude variation

The average output amplitude variation:

5 $${\sigma }_{\alpha =0}={[\frac{\sigma ({y}_{n+1}^{out})}{\sigma ({y}_{n+1}^{T})}]}^{2},$$

where *n* = 2010, 2011, …, 2035.

### Standard deviation

The standard deviation of all node responses individually averaged over time is measured against the rRNN’s mean-field dynamical state:

6 $${\delta }_{n+1}=\frac{1}{\mu }\sqrt{\frac{1}{N}\sum _{i=1}^{N}{({x}_{n+1}^{i})}^{2}-{(\frac{1}{N}\sum _{i=1}^{N}{x}_{n+1}^{i})}^{2}},$$

with i∈ [1, 500]. A normalization by *μ* then allows to associate *δ*_*n*+1_ to a synchronization error in phase of the nonlinear function in Eq. (1).

### The Mackey-Glass system

**Figure 6:**
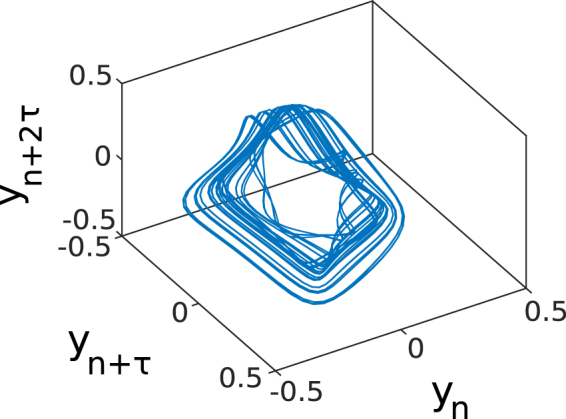
3D projection of the MG attractor.

The MG system is a first order nonlinear delay differential equation^32^, whose time-discrete version is the following^33^:

7 $${y}_{n+1}={y}_{n}+\delta (\frac{0.2{y}_{{\tau }_{m}}}{1+{({y}_{{\tau }_{m}})}^{10}}-0.1{y}_{n}),$$

where ${y}_{{\tau }_{m}}=y(n-{\tau }_{m}/\delta )$, *τ*_*m*_ = 17 as the time delay, and *δ* = 1/10 is the stepsize indicating that the time series is subsampled by 10. Where 1 increment of the resultant time series correspond to 10 time units of the Mackey-Glass equation. The MG time series is shifted to oscillate around zero. For the reconstruction of the attractor we apply Takens embedding Theorem^34^ to the time series *y*_*n*+1_. We used embedding parameters of delay *τ* = 12 and dimensions *D* = 4 which are the ones obtained for delay embedding the original MG attractor^35^. Figure 6 shows a 3D projection of the MG attractor.

### Mutual information

Mutual information quantifies the amount of information shared between node responses ${x}_{n+1}^{i}$ and input signal ${y}_{n+1}^{T}$^39^

8 $$M{I}_{i}=\sum _{{x}_{n+1}^{i},{y}_{n+1}^{T}}{\mathscr{P}}({x}_{n+1}^{i},\,{y}_{n+1}^{T})\,\mathrm{log}\,\frac{{\mathscr{P}}({x}_{n+1}^{i},{y}_{n+1}^{T})}{{\mathscr{P}}({x}_{n+1}^{i}){\mathscr{P}}({y}_{n+1}^{T})}\mathrm{.}$$

Mutual information *MI* therefore depends on the joint probability density function of ${x}_{n+1}^{i}$ and ${y}_{n+1}^{T}$, ${\mathscr{P}}({x}_{n+1}^{i},\,{y}_{n+1}^{T})$, as well as ${\mathscr{P}}({x}_{n+1}^{i})$, ${\mathscr{P}}({y}_{n+1}^{T})$ which are the probability density functions of *x*^*i*^ and *y*^*T*^, respectively. If *MI*_*i*_ >> 0 for the *i*th node, it preserves most dynamical properties of input ${y}_{n+1}^{T}$. Under these conditions the rRNN as a whole therefore is capable to preserve the input information without significant loss of information, hence learning should be possible in principle. By accumulating all *MI*_*i*_ in the global measure *C* = ∑_*i*_(*MI*_*i*_) defined as memory capacity^26^.

### Maximal Lyapunov exponent

Complex dynamical systems are typically classified using the rate of exponential divergence between neighbor trajectories, corresponding to their Lyapunov exponent. Specifically chaotic systems have a positive maximal Lyapunov exponent *λ*_*max*_^40^. The maximal Lyapunov exponent^40–42^ is at first calculated for each *i*-th node, ${\lambda }_{max}^{i}$. Then the maximal Lyapunov exponent of the rRNN is ${\lambda }_{max}=\,{\rm{\max }}({\lambda }_{max}^{i})$.
